# Bicycle Rider Behavior and Crash Involvement in Australia

**DOI:** 10.3390/ijerph18052378

**Published:** 2021-03-01

**Authors:** Steve O’Hern, Nora Estgfaeller, Amanda N. Stephens, Sergio A. Useche

**Affiliations:** 1 Monash University Accident Research Centre, Clayton 3800, Australia; nora.estgfaeller@monash.edu (N.E.); amanda.stephens@monash.edu (A.N.S.); 2 Transport Research Centre Verne, Tampere University, 33014 Tampere, Finland; 3 Faculty of Psychology—INTRAS Research Centre, University of Valencia, 46022 Valencia, Spain; sergio.useche@uv.es

**Keywords:** cycling, behaviour, road safety

## Abstract

This research investigated how behaviours and attitudes of bicycle riders influence crash frequency and severity. The study recruited 1102 Australian bicycle riders for an online survey. The survey comprised questions on demographics, frequency of riding and the number and severity of traffic crashes during the last five years. The survey included the Cycling Behaviour Questionnaire and the Cyclist Risk Perception and Regulation Scale. Overall, there were low levels of errors and violations reported by participants indicating that these behaviours were on average never or rarely exhibited while riding a bicycle. Conversely, participants reported high levels of engagement in positive behaviours and reported high levels of traffic rule knowledge and risk perception. Higher rates of violations and errors were associated with increased crash likelihood, while higher rates of positive behaviours were associated with reduced rates of crash involvement in a period of 5 years. The findings highlight the relationship between errors, total crashes and crash severity Further promotion of positive behaviours amongst riders may also help to reduce the risk of crashes.

## 1. Introduction

Shutdowns experienced during the COVID-19 pandemic have seen travel patterns change throughout the world [1,2,3]. In particular, recent studies have shown that the level of public transport use may take some time to return to pre-COVID levels, especially due to health and safety concerns of commuters [3,4,5].

This means that many commuters will adopt other modes of transport, or prefer to drive, leading to increased congestion on the roads. To avoid such congestion, there is a need to promote bicycle riding amongst a suite of sustainable transport options [6]. Indeed, cycling can be undertaken at safe social distances [7]. Many cities have responded by encouraging increased active transportation through the reallocation of urban space, for example by installing ‘pop-up- bicycle lanes’ [6] and there is recognition that this increase in active transport usage can continue post-pandemic [6,8]. These changes in transport patterns have the potential to maintain positive long-term impacts on mobility. However, in order to maintain and increase the number of people riding bicycles, there is a need to improve safety particularly when riding on-road. Road user behaviours are one issue to address to assist in achieving this goal.

The main risk associated with riding a bicycle on-road is being hit by a motor vehicle. In fact, this potential danger is a key deterrent against riding a bicycle [9,10], particularly amongst less experienced bicycle riders and people with lower tolerance to risk. Therefore, in order to facilitate growth in the number of people riding bicycles, safety issues need to be addressed [11]. This is especially the case for on-road riding [11,12,13,14] with higher traffic volumes and vehicular speeds, which increases the injury risk in the event of a crash [15,16]. Somewhat paradoxically, as the number of people riding bicycles increases, the population risk for riders decreases [17], a fact that is evidenced by the substantially lower injury rates for bicycle riders in the Netherlands and Denmark, two nations with high levels of cycling participation [18]. Furthermore, the risk associated with riding a bicycle, is outweighed by the benefits for the community and the individual [19,20,21], providing increased justification to promote bicycle riding as a sustainable transportation option.

In Australia, cyclists are over-represented in injury statistics [22,23] and cycling trauma represents a major transport safety issue [24]. Furthermore, despite a climate that is favourable to active transportation [25], cycling mode share remains relatively low [26], and participation has declined in some areas. Analysis of injury statistics identifies key injury-crash risks including riding at night, on roads with higher speed limits, in rural areas, and while under the influence of alcohol and illicit substances [22], therefore highlighting that the behaviours, either intentional or unintentional, of some people while riding increases their crash risk. Likewise, the behaviour of various road user groups (such as private vehicle drivers, people riding motorcycles, taxi drivers as well as people riding bicycles) is also a contributing factor for crashes [27,28,29,30,31,32]. Understanding the relationships between bicycle rider behaviour and crash risk is therefore important in order to improve road safety and increase bicycle riding uptake.

Research in the driving domain has shown two distinct types of aberrant behavior—errors and violations—which have different underlying mechanisms [33]. Errors reflect unintentional mistakes and are due to the information processing characteristics of the road user. Conversely, violations are deliberate behaviours that contravene road rules and are explained by social and motivational factors [33]. Building on the work of Reason et al. [33] researchers have extended these constructs from driving to other road user groups, including bicycle riders [34,35,36,37].

Recently, Useche [38] validated a Cycling Behaviour Questionnaire (CBQ), based on an international cohort of 1064 bicycle users from 20 countries. In line with the findings from Reason, Useche [38] identified key dimensions of violations and errors that describe risky behaviours that people engage in while riding a bicycle. Useche [38] also identified a third dimension describing positive behaviours, a dimension that has also been noted in recent versions of the Driving Behaviour Questionnaire [39]. Using the validated CBQ, recent studies have investigated the association between risky behaviours (errors and violations combined) and crashes while riding a bicycle.

Zheng [40] analysed the self-reported crashes in a period of 12 months, finding that Chinese riders’ crash risk can be directly predicted by various factors, with risky cycling behaviors measured through the CBQ its strongest predictor. Another study used the past five years as time frame and found that people who engaged more often in risky behaviours while riding a bicycle also tended to report higher crash involvement [41]. While a significant relationship between crashes and risky behaviours was identified, this was only moderate, suggesting that there are other factors, such as risk perception and rule knowledge, potentially contributing to crash involvement. Furthermore, while risky behaviours may not contribute directly to crash risk, they may have an indirect effect, for example making avoidance manoeuvres more complicated to perform. This aligns with previous research that identified amongst drivers that engagement in risky behaviours themselves is not a strong predictor of all crash types [42].

By a similar logic, positive behaviours do not totally exempt a road user from risk or reflect a lack of risk taking [39]; however, positive behaviours have been shown to be negatively associated with errors and violations and have a protective effect [39]. Engagement in positive and risky behaviours are influenced by road user’s attitudes and awareness of road traffic rules and situations [43], for example, their knowledge of traffic norms or their risk perception [44]. Attitudes represent more immediate precursors of self-reported intentions than behaviours [45] and represent affective evaluations towards a specific object [46]. Research has demonstrated that attitudes towards traffic safety can help to mitigate risky behaviours amongst drivers [43] and that road safety knowledge can be a protective factor for road traffic crashes. Conversely, road users with negative attitudes towards traffic safety are more likely to have a high propensity for risky behaviours [47].

However, while research addressing road user behaviour and attitudes is common amongst motor vehicle drivers, studies on attitudes and behaviours of bicycle riders are scarce. In a study of Colombian, Mexican and Argentinian bicyclists, Useche [44] identified that knowledge of traffic norms and higher risk perception were protective factors and were associated with reduced risky behaviours and an increase in positive behaviours. However, research has also demonstrated that the behaviour and attitudes of people riding bicycles differ by jurisdiction; for example, Useche [48] demonstrated differences when considering Latin American, European and North American bicycle riders’ cycling patterns and behavioural outcomes. Similarly, Oehl [49] demonstrated significant differences in self report bicycle rider anger when comparing results from Australia, Singapore, China and Germany.

To our knowledge, positive behaviours, and attitudes regarding knowledge of traffic norms and risk perception are yet to be investigated amongst an Australian cohort of riders, in order to understand their effects on crash involvement. In practical terms, further understanding these relationships in different locations will help to inform safety interventions and understand the nuances in bicycle rider behaviours. As such, the core aim of this research was to investigate how these factors influence crash quantity and severity amongst a cohort of Australian bicycle riders. The expectation is that through understanding bicycle rider behaviours and attitudes, interventions can be developed to reduce engagement in risky behaviours, increase positive behaviours, and ultimately increase the number of people riding bicycles for transportation.

## 2. Methods

### 2.1. Procedure

Data were collected via an online survey using a paid advertisement on Facebook. Advertising of the survey occurred for four weeks in July 2020. This period corresponded with COVID19 restrictions being in effect across most of Australia. Participants were provided with an explanatory statement outlining the format of the survey and that they could complete the survey in their own time. The survey took approximately 20 min to complete and was incentivised with the chance to win an AUD$200 voucher. In order to guarantee the anonymity of their data, participants opted into the draw for the voucher via a separate link provided upon completing the survey. Ethical approval for the study was obtained from the University Human Research Ethics Committee.

### 2.2. Participants

A total of 1408 participants started the online survey, with 1102 completing all items, representing a completion rate of 78.3%. To be eligible to participate in the study, respondents needed to indicate that they resided in Australia, were over the age of 18, had ridden a bicycle in the past year and were proficient in English. Of the 1102 respondents 29.0% (320) identified as female, 70.1% (772) identified as male and 0.9% (10) identified as non-binary. The participants’ age ranged between 18 and 80, with a mean age of 50.4 (SD = 12.7) years. The higher proportion of male participants and average age are in line with previously reported statistics on the population of Australians who ride bicycles [50,51]. For example, the most recent national cycling participation survey identified that on a weekly basis 17.3% of Australian males ride a bicycle compared to 10% of females, with weekly participation rates declining in all age groups except those over 50 [26].

Participants were located throughout Australia with responses recorded from each state and territory. The highest response rates were from Victoria (34.4%) New South Wales (22.3%) and Queensland (14.2%), followed by Western Australia (9.0%), South Australia (7.9%) and Tasmania (6.3%). The distribution of responses demonstrated a good correlation with population statistics (r = 0.88, *p* = 0.004) [52].

Almost three quarters (74.4%) of participants held a university degree (36.9% undergraduate, 37.5% postgraduate), 200 (18.1%) respondents had received technical training and 82 (7.4%) stated a high school diploma as their highest level of education. The majority (72.7%) were either employed or self-employed. Of the remaining participants, 181 (16.4%) were retired, 50 (4.5%) were students, 46 (4.2%) were unemployed and 24 (2.2%) had another main occupation.

Participants reported that typically they rode a bicycle for 10.3 h (SD = 7.7) per week. When asked about crash involvement while cycling over the past five years, 67.8% of participants reported having suffered at least one riding crash (M = 3.2, SD = 7.5), with 28.2% of participants requiring medical attention as a result of the injuries they sustained.

### 2.3. Materials

The survey comprised questions on demographic characteristics, frequency of riding and the number and severity of traffic crashes while riding a bicycle during the last five years. The survey also included: the Cycling Behaviour Questionnaire (CBQ) [38] and the Cyclist Risk Perception and Regulation Scale (RPRS) [41].

#### 2.3.1. Cyclist Behaviour Questionnaire (CBQ)

Self-reported behaviours were assessed using the Cycling Behaviour Questionnaire (CBQ) [37]. The CBQ follows the factor structure developed by Reason [33] for the Driving Behaviour Questionnaire (DBQ) and includes two factors measuring risky behaviours (errors, that are unintended risky behaviours and violations, that unlike errors, are deliberate behaviours). A third factor, positive behaviours, is included in this version of the CBQ. Positive behaviours represent protective factors for the occurrence of crashes. The CBQ comprised 29 items representing the three factors: Violations, consisting of 8 items; Errors, 15 items; and Positive Behaviours 6 items. For each item, participants indicated how often, if at all, they perform each behaviour while riding a bicycle. Responses were recorded on a five-point Likert scale: 1 = never, 3 = sometimes and 5 = almost always. Previous applications of this version of the CBQ have demonstrated good internal consistency and reliability, with Cronbach’s alphas ranging from 0.70 to 0.85 and composite reliability indexes (CRIs) ranging between 0.80 and 0.90 [38].

#### 2.3.2. Cyclist Risk Perception and Regulation Scale (RPRS)

Risk perception and knowledge of traffic regulations while cycling were measured using the Cyclists Risk Perception and Regulation Scale (RPRS) [41]. The RPRS is a 12-item scale measuring risk perception (7 items) and knowledge of traffic rules for bicycle use (5 items). Responses were given on a five-point Likert scale from 1 = strongly disagree, 3 = neither agree nor disagree, and 5 = strongly agree. The RPRS has demonstrated acceptable internal consistency with Cronbach’s alphas ranging from 0.66 to 0.72 [41].

### 2.4. Analysis

Data were analysed using IBM © SPSS v.25 with alpha (α) set to 0.05. Only complete responses were included in the analysis, and as such there were no missing data. Pearson’s correlation coefficients identified relationships between demographic, average weekly time riding a bicycle, factors in the CBQ and RPRS and total crashes. Multivariate Analysis of Covariance (MANCOVA) was conducted with crash type as a fixed factor. The factors from the CBQ and RPRS were included as dependent variables with age and average weekly time riding a bicycle included as covariates. Model fit was assessed with Wilks’ Lambda (Λ) with effect sizes reported as partial eta squared (η^2^). Finally, multinomial logistic regression (MLR) tested the role of the CBQ and RPRS factors on crash severity while cycling over the past five years. Due to the low number of non-binary respondents, these participants were not included in the multinomial logistic regression. Goodness of fit was assessed using Nagelkerke pseudo R^2^ with exponential coefficients reported as odds ratios.

## 3. Results

Table 1 and Table 2 summarise the mean and standard deviation of items for each factor for the CBQ and RPRS respectively. The reliability of the factors from the CBQ and RPRS were assessed using Cronbach’s alpha to confirm the internal consistency of the items loading onto each factor. Cronbach’s alpha was lower than previously reported in the literature [38,41]. However, overall reliability scores for each factor, ranging from 0.65–0.86, were acceptable.

Low mean scores were reported for all violations included in the CBQ. The lowest mean score was for carrying passengers on a bicycle, with most respondents (95.7%) reporting that they never engage in this behaviour. The most common violation was travelling faster than one should, with only 27.3% of respondents stating that they never do so while riding a bicycle.

Mean scores for errors were also low, indicating that participants assess these as relatively rare events. The most common error was braking abruptly on a slippery surface, with 62.5% of respondents reporting this behaviour occasionally. Unlike traffic violations and errors, mean scores for positive behaviours were typically high, indicating frequent engagement in these behaviours. The highest mean score was for using a path or bike lane when one is available. The lowest mean scores were found for two protective habits, i.e., avoiding riding during adverse weather conditions, or when feeling sick or tired.

High mean scores were reported for all items in the RPRS indicating that participants had good knowledge of traffic rules and good self-reported risk perception. The lowest mean score was regarding pedestrian priority, with only 34.7% agreeing (4) or strongly agreeing (5) that pedestrians should always have priority. On average, participants strongly agreed with all other items.

Table 3 presents individual relationships amongst demographic variables, weekly time spent riding a bicycle, total crashes in the past five years and the CBQ and RPRS factors. Significant negative relationships were found between age, violations and total crashes. Conversely, significant positive relationships were found for positive behaviours, knowledge of traffic rules and risk perception when compared to age. The number of hours spent riding a bicycle per week demonstrated a significant positive correlation with violations and errors. However, weekly cycling was also negatively correlated with positive behaviours. Unsurprisingly, increased cycling was associated with increased crashes and this is likely to be a reflection of increased exposure. Total crashes were positively associated with errors, violations and negatively associated with positive behaviours. No significant relationship was identified between risk perception and knowledge of traffic rules and total crashes.

**Figure 1:**
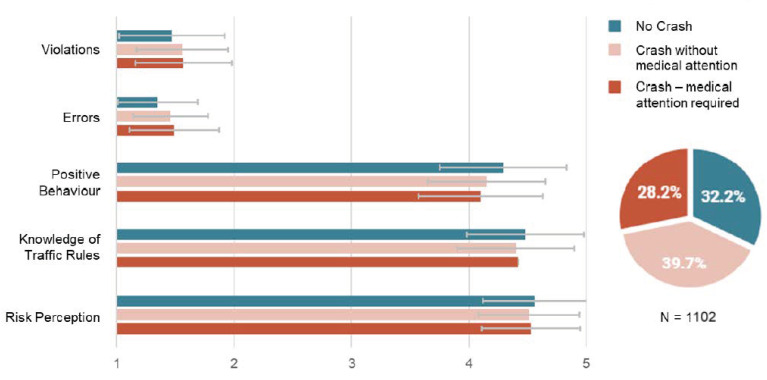
Summary of Cycling Behaviour Questionnaire (CBQ) and Risk Perception and Regulation Scale (RPRS) by crash involvement.

Figure 1 provides a summary of the differences in crash involvement when considering the factors of the CBQ and RPRS. The MANCOVA (presented in Table 4) had a Wilks’ Lambda of 0.962 (F _(10)_ = 4.23, *p* ≤ 0.001) indicating that the overall main effect of crash involvement reached significance. Across the sample of participants, 355 (32.2%) reported no crashes while cycling over the past 5 years, 438 (39.7%) reported only minor crashes that required no medical treatment, while the remaining 311 (28.2%) reported being involved in at least one crash that required medical treatment, either at a hospital, by paramedics or from a doctor or nurse.

In total, there were low levels of errors (1.43) and violations (1.53) reported by participants indicating that these behaviours were on average never or rarely exhibited while riding a bicycle. Conversely, participants reported high levels of engagement in positive behaviours while cycling (4.18) and reported high levels of traffic rule knowledge (4.43) and risk perception (4.53). Significant differences were identified for violations, errors and positive behaviours when considering crash involvement, with higher rates of violations and errors associated with increased crash involvement (*p* ≤ 0.01 and *p* = 0.012) and higher rates of positive behaviours associated with reduced rates of crash involvement (*p* ≤ 0.01). However, while the differences were statistically significant, the effect size was small and only small differences in mean factor scores were observed. No significant relationship was identified between crash involvement and self-reported knowledge of traffic rules or risk perception. The covariates age (Λ = 0.933, F (5) = 16.5, *p* ≤ 0.001) and weekly hours cycling (Λ = 0.957, F (5) = 11.0, *p* ≤ 0.001) were significant.

Multi-nominal logistic regression (MLR) was undertaken to examine the relationship between crash type and involvement and the CBQ and RPRS factors (Table 5). The model identified a significant relationship between gender and crash severity, with males reporting a significantly higher rate of involvement in crashes that did not require medical attention (OR = 1.9; 95%CI: 1.39, 2.66), however, the finding was not statistically significant when considering crashes requiring medical attention (OR = 1.4; 95%CI: 1.00, 2.00, *p* = 0.053). Notwithstanding, the analysis highlights the increased likelihood of males self-reporting minor injury crashes and those requiring medical attention compared to females. Interestingly, age was not a significant predictor of crash severity, despite the correlation analysis identifying a significant negative relationship between total crashes and age. Of the factors included in the CBQ, the odds of minor crashes and crashes requiring medical attention increased with higher rates of self-reported errors, while there was a protective factor associated with positive behaviours, but only for crashes requiring medical attention. Neither knowledge of traffic rules, nor risk perception were associated with crash severity.

## 4. Discussion

The aim of this study was to investigate the associations between self-reported bicycle riding behaviours, attitudes regarding risk perception, knowledge of traffic rules and the frequency and severity of self-reported crashes amongst Australian cyclists. This was done using two previously validated measures designed for bicycle riders; the CBQ, measuring behaviour [38] and the RPRS, measuring risk perception and road rule knowledge [41]. The results showed that behaviours were significantly associated with crash outcomes. Specifically, high levels of errors and low levels of positive behaviours were associated with crashes, albeit generally participants reported low levels of risky behaviours.

Overall, people in the sample reported low levels of errors and violations while riding a bicycle. These findings are similar to previous applications of the CBQ and similar self-report-based assessment tools worldwide, including Useche [38], Zheng [40], Feenstra [35], Hezaveh [53] and O’Hern [30], who found that people who ride bicycles tend to exhibit low levels of risky behaviours. Despite the infrequency of these behaviours, correlation analysis showed a weak positive relationship between self-reported risky behaviours and total crashes over the past five years, demonstrating that cyclists who engage in errors or violations more often also tend to report a higher number of crashes, as consistently found across the aforementioned studies. Further, there was some evidence that the frequency of errors was also related to the severity of the crashes. After controlling for age, gender, cycling frequency and attitudes, the odds of crash involvement (minor or injury) increased as self-reported error frequency increased. The findings align with research by Hezaveh [53], who identified that control errors and notice failures were significantly associated with self-reported crash involvement. This may be because errors are unintentional behaviours which are beyond the control of the rider and can occur at any time, increasing their risk of being involved in a crash. On the other hand, violations are deliberate behaviours and as such riders may choose when to engage in them (i.e., when they feel it is safe to do so) [30]. At the same time, cyclists are often not at fault when involved in a collision, particularly when other road users are involved [54], and this may explain the relatively low predictive power of behaviours for crash involvement for cyclists.

The third factor in the CBQ considered cyclists’ positive behaviours. Generally, participants self-reported high levels of positive behaviours and there was a negative relationship between positive behaviours and total crashes, suggesting these behaviours may be a protective factor for crash risk. This was demonstrated with a small, but statistically significant difference between crash involvement and positive behaviours with higher average self-reported positive behaviours amongst cyclists who had not had a crash over the past 5 years. Similarly, increased positive behaviours were associated with a significantly lower rate of severe crashes in the multinomial logistic regression when controlling for other variables. Also, as with previous research investigating positive behaviours amongst drivers [39], there was a negative association between risky behaviours and positive behaviours. While engagement in positive behaviours does not exempt cyclists from risk, the research has demonstrated the potentially protective benefits of engaging in positive behaviours and that they should be promoted amongst bicycle riders [5]. Furthermore, there are avenues for further research into positive behaviours when riding bicycles, such as the use of protective equipment.

When considering cyclists’ knowledge of traffic rules and risk perception, as expected, there were negative relationships with errors and violations, indicating that higher knowledge of traffic rules and better self-reported risk perception were associated with reductions in risky behaviours. Furthermore, those with a high perception of risk would be less likely to engage in risky behaviours. However, across the cohort, all participants reported high knowledge of traffic rules and risk perception and there was minimal variation between those involved in crashes. As such, the predictive capacity of these variables may not have been realised in this cohort of participants, who were generally older and rode frequently. Notwithstanding, the findings align with previous applications of the RPRS by Useche et al. [44]. In this study considering older cyclists in Latin America it was identified that risk perception and knowledge of traffic rules were not direct predictors of traffic crashes; however, Useche [44] did identify a significant relationship between knowledge of traffic rules and risk perception and engagement in risky behaviours, highlighting their mediating role in risky behaviours. The findings also align with research by Wang, who identified a significant relationship between risk perception and behaviour amongst young cyclists in China [55].

Another important finding was the significant gender difference in self-reported crashes. The finding aligns with previous research that has demonstrated females have higher risk perception and lower risk profiles than males when riding a bicycle [56,57]. Conversely, males have a greater propensity for risk taking behaviours compared to female cyclists [58] and are also more likely to demonstrate aggressive behaviours while cycling, which is associated with increased crash risk [51] and may contribute to their increased exposure. However, the protective effect of gender did not result in a significant reduction in crashes requiring medical attention. This may demonstrate the increasingly complex nature of these collisions and the reduced ability for riders to influence these more serious crashes.

A negative relationship was identified between age and total crashes in the previous five years, indicating that older riders tended to report less crashes. This is an interesting finding, as previous analysis of cyclist injuries in Australia has indicated that older males are over-represented in injury statistics [22,23]; however, it is noted that the study recruited a relatively older cohort of participants and these findings need to be confirmed on a larger and more diverse sample. Notwithstanding this limitation, while older persons may be less likely to be involved in a crash, they are increasingly likely to suffer more serious injuries, which is linked to increased physical frailty [59]. Age was related to an increase in positive behaviours, risk perception and knowledge of traffic rules and a reduction in self-reported risky behaviours. These findings are in line with previous research that identified similar trends in Latin America, Europe and North America [41,44]. Similarly, Schleinitz [60] found that age also modulated risk-related and decision-making judgments, in their study interviewing riders. Ecological research by Martinez-Ruiz [61] demonstrated the relationship between mortality, risk and frailty and highlights similar trends in survey findings, with younger riders having a greater risk of crash involvement while the higher rates of mortality for older riders was associated with physical frailty rather than, e.g., risk assumption and reckless behaviour, that are more frequently observed among younger riders.

Interestingly, those who reported increased weekly time spent riding a bicycle had higher rates of violations and errors and reductions in positive behaviours. However, this may reflect a greater opportunity to perform aberrations with increased exposure; again, Martinez-Ruiz notes the relationship between exposure and crash risk, particularly for younger persons [61].

### Limitations

There are several noted limitations with this research. The recruitment process for the study required participants to be over the age of 18 years and be regular riders of a bicycle on-road. This will have excluded younger riders and will limit the generalisability of the findings to those who do not ride bicycles regularly. While the sample was drawn from a diverse geographic region, the study was biased towards older cyclists and there was an over-representation of males. However, these demographics are more commonly associated with cycling in Australia [26]. Furthermore, it is worth acknowledging that this study was based on an online survey, and as such certain segments of the population (whose interaction to connected devices may be less) could be under sampled in the study.

Another noted limitation of this study is that the findings are based on self-reported survey data. This has likely to have introduced a self-selection bias in the sample, with those with a strong interest in cycling more likely to participate. Similarly, self-report may introduce social desirability bias when responding. However, to counteract this, all potential participants were assured that participation was voluntary, and their responses were anonymous. The study highlights the relationship between crashes and rider behaviour; further understanding of collision mechanisms and the specifics of crashes through in-depth investigations would provide greater understanding of the crash risk factors. Similarly, the behaviours exhibited by riders could be objectively measured and validated using observational or naturalistic study designs.

## 5. Conclusions

The findings from this study highlight key road safety issues for bicycle riders. As with previous research, we found that self-reported errors and violations are rare and that bicycle riders typically have high levels of knowledge regarding road rules.

Further promotion of positive behaviours amongst cyclists may also help to reduce the risk of crashes, particularly amongst new riders who have recently adopted this sustainable mode of transport. Of course, there is recognition that many crashes are not the fault of cyclists and that most risk cannot be resolved through addressing bicycle rider behaviours. Further research on interaction between drivers and bicycle riders is needed and measures that improve safety such as dedicated infrastructure and lower motor vehicle speeds should be promoted alongside programs that promote positive bicycle rider behaviours. In this regard, this study is one of the first addressing positive behaviour of cyclists in Australia, apart from the risky riding behaviours (errors and violations) that are traditionally measured in similar studies.

## Figures and Tables

**Table 1:** Cyclist behaviour questionnaire (CBQ) item scores (scale 1–5).

Factor (Cronbach’s Alpha)	Item	M (SD)
Violations (0.69)	Cycling under the influence of alcohol and / or other drugs or hallucinogens	1.34 (0.67)
	Riding against the traffic flow (wrong way)	1.43 (0.70)
	Zigzagging between vehicles when using a mixed lane	1.53 (0.81)
	Handling potentially obstructive objects while riding a bicycle (food, packages, etc.)	1.54 (0.83)
	Feeling that sometimes I’m going at a higher speed than I should be	2.06 (0.83)
	Crossing what appears to be a clear crossing, even if the traffic light is red	1.88 (0.93)
	Carrying a passenger on my bicycle without it being adapted for such a purpose	1.06 (0.30)
	Having a “race” with another cyclist or driver	1.42 (0.73)
	Unintentionally crossing the street without looking properly, thus making another vehicle brake to avoid a crash	1.25 (0.50)
	Colliding (or being close to it) with a pedestrian or another cyclist while cycling distractedly	1.32 (0.54)
	Braking suddenly and being close to causing an accident	1.52 (0.63)
Error (0.86)	Failing to notice the presence of pedestrians crossing when turning	1.37 (0.56)
	Not braking at a “Stop˜ or ˜Give Way” sign and being close to colliding with another vehicle or pedestrian	1.30 (0.58)
	Braking very abruptly on a slippery surface	1.77 (0.70)
	While I am distracted, I do not realise that a pedestrian intends to cross a crosswalk, and therefore I do not stop to let them do so	1.33 (0.57)
	Not realising that a parked vehicle intends to leave and consequently having to brake abruptly to avoid a collision	1.80 (0.76)
	When riding on the left side, not realising that a passenger is getting out of a vehicle or bus, and thus being close to hitting them	1.52 (0.71)
	Trying to overtake a vehicle that had previously used its indicators to signal that it was going to turn, consequently having to brake	1.39 (0.67)
	Misjudging a turn and hitting something on the road, or being close to losing balance (or falling)	1.70 (0.69)
	Unintentionally hitting a parked vehicle	1.08 (0.34)
	Failing to be aware of the road conditions and falling over a bump, hole or obstacle	1.67 (0.71)
	Confusing one traffic signal with another, manoeuvring according to the latter	1.19 (0.45)
	Trying to brake but not being able to use the brakes properly due to a poor hand positioning	1.28 (0.53)
Positive Behaviour (0.65)	I stop and look at both sides before crossing a corner or intersection	4.40 (0.95)
	I try to move at a prudent speed to avoid sudden mishaps or braking	4.31 (0.84)
	I usually keep a safe distance from other cyclists or vehicles	4.48 (0.66)
	When I use the bike path (or bike-lane), I always use the indicated lane	4.55 (0.68)
	I avoid cycling under adverse weather conditions	3.53 (1.06)
	I avoid cycling if I feel very tired or sick	3.84 (1.05)

**Table 2:** Cyclists Risk Perception and Regulation Scale (RPRS) item scores (scale 1–5).

Factor (Cronbach’s Alpha)	Item	M (SD)
Knowledge of Traffic Rules (0.62)	I readily recognise traffic signals	4.78 (0.65)
	I know the basic rules governing other types of vehicles	4.77 (0.61)
	I believe that pedestrians should always have the priority, even towards cyclists	3.85 (1.09)
	I easily identify areas prohibited to traffic or bicycle parking	4.23 (0.86)
	Overall, I know the bicycle safety regulations of my city/town	4.52 (0.68)
	I am aware of the potential consequences of being involved in a crash, for example, with another vehicle	4.67 (0.68)
	I perceive potentially higher risks for my integrity when I ride a bicycle, than when I am on board of a motorised vehicle	4.51 (0.82)
Risk Perception (0.68)	I am always aware of the other vehicles that surround me on the road	4.53 (0.62)
	I realise that there are signalling and infrastructure problems that can affect my safety	4.50 (0.65)
	I believe that being under the influence of certain substances (alcohol, illegal and/or prescribed drugs) affects my ability to ride well	4.74 (0.58)
	I am aware of the risks involved in using headphones and phones while I ride	4.60 (0.77)
	Riding in urban areas is especially risky, considering the number of vehicles and the complexity of the roads	4.17 (0.99)

**Table 3:** Bivariate (Pearson) Correlations.

	Factor	1	2	3	4	5	6	7	8
1	Age	1							
2	Weekly cycling (hours)	0.048	1						
3	Violations	−0.210 **	0.179 **	1					
4	Errors	−0.050	0.177 **	0.510 **	1				
5	Positive Behaviours	0.169 **	−0.133 **	−0.394 **	−0.241 **	1			
6	Knowledge of Traffic Rules	0.135 **	−0.032	−0.259 **	−0.236 **	0.352 **	1		
7	Risk Perception	0.165 **	−0.021	−0.331 **	−0.169 **	0.419 **	0.492 **	1	
8	Total Crashes	−0.065 *	0.100 **	0.078 *	0.071 *	−0.106 **	−0.001	0.009	1

Notes: ** *p* ≤ 0.01, * *p* ≤ 0.05.

**Table 4:** MANCOVA of CBQ and RPRS factors and crash severity.

Factor	Total	No Crash	Crash—No Medical Attention Required	Crash—Medical Attention Required	F	η^2^
Violations	1.53 (0.42)	1.47 (0.45)	1.56(0.39)	1.57 (0.41)	4.46 *	0.01
Errors	1.43 (0.36)	1.35(0.37)	1.46 (0.32)	1.49 (0.38)	13.12 **	0.02
Positive Behaviours	4.18 (0.54)	4.29 (0.54)	4.15 (0.52)	4.10 (0.53)	11.03 **	0.02
Knowledge of Traffic Rules	4.43 (0.50)	4.48 (0.50)	4.40 (0.50)	4.42 (0.50)	2.90	0.01
Risk Perception	4.53 (0.44)	4.56 (0.45)	4.51 (0.43)	4.53 (0.42)	1.26	0.00

Notes: Wilks’ Lambda (Λ) = 0.962, F = 4.23 *p* ≤ 0.001. Age and weekly distance riding a bicycle included as covariates. **. *p* ≤ 0.01, *. *p* ≤ 0.05.

**Table 5:** Multinomial logistic regression model results for crash involvement.

Parameter	Crash—No Medical Attention Required				Crash—Medical Attention Required			
	Β	Std. Error	OR	95% CI of OR	Β	Std. Error	OR	95% CI of OR
Age	−0.004	0.006	0.996	0.985–1.008	0.012	0.007	1.012	0.999–1.025
Gender (Male)	0.654 **	0.166	1.923	1.389–2.663	0.344	0.178	1.410	0.995–1.998
Weekly cycling (hours)	0.013	0.012	1.013	0.990–1.036	0.024	0.012	1.024	1.001–1.048
Violations	−0.197	0.233	0.821	0.520–1.295	−0.128	0.250	0.880	0.539–1.436
Errors	1.093 **	0.281	2.982	1.718–5.177	1.209 **	0.298	3.349	1.869–6.002
Positive Behaviours	−0.293	0.170	0.746	0.534–1.042	−0.575 **	0.181	0.550	0.386–0.785
Knowledge of traffic rules	−0.158	0.174	0.854	0.607–1.201	−0.038	0.192	0.962	0.661–1.402
Risk perception	0.096	0.210	1.101	0.730–1.661	0.229	0.230	1.257	0.800–1.974
(Intercept)	0.089	1.230			−1.063	1.339		

Notes: Base category no crashes. Likelihood χ^2^ = 81.2 **, Nagelkerke Pseudo R^2^ = 0.081. **. *p* ≤ 0.01.

## Data Availability

The data presented in this study are available on request from the corresponding author. The data are not publicly available due to privacy restrictions.
